# Type D Personality Is Associated with Psychological Distress and Poor Self-Rated Health among the Elderly: A Population-Based Study in Japan

**DOI:** 10.1371/journal.pone.0077918

**Published:** 2013-10-17

**Authors:** Yosuke Kasai, Etsuji Suzuki, Toshihide Iwase, Hiroyuki Doi, Soshi Takao

**Affiliations:** 1 Department of Epidemiology, Graduate School of Medicine, Dentistry and Pharmaceutical Sciences, Okayama University, Okayama, Japan; 2 Support Center for Medical Cooperation, Human Resource Placement and Career Promotion of Okayama Prefecture, Graduate School of Medicine, Dentistry and Pharmaceutical Sciences, Okayama University, Okayama, Japan; Federal University of Rio de Janeiro, Brazil

## Abstract

We investigated the association between Type D personality, psychological distress, and self-ratings of poor health in elderly Japanese people. In August 2010, questionnaires were sent to all residents aged ≥65 in three municipalities (*n* = 21232) in Okayama Prefecture, Japan, and. 13929 questionnaires were returned (response rate: 65.6%). To assess mental and physical health outcomes, we used the Kessler Psychological Distress Scale and a single item question regarding perceived general health. We analyzed 9759 questionnaires to determine odds ratios (ORs) and 95% confidence intervals (CIs) for several health outcomes, adjusting for sex, age, smoking status, frequency of alcohol consumption, overweight status, educational attainment, socioeconomic status, and number of cohabiters. The multiple imputation method was employed for missing data regarding Type D personality. The prevalence of Type D personality in our sample was 46.2%. After adjusting for covariates, we found that participants with Type D personality were at 4–5 times the risk of psychological distress, and twice the risk of poor self-rated health. This association was stronger in participants aged 65–74 years (psychological distress; OR: 5.80, 95% CI: 4.96–6.78, poor self-rated health; OR: 2.84, 95% CI: 2.38–3.38) than in those aged over 75 years (psychological distress; OR: 4.54, 95% CI: 3.96–5.19, poor self-rated health; OR: 2.05, 95% CI: 1.79–2.34). Type D personality is associated with adverse health status among Japanese elderly people in terms of mental and physical risk; therefore, further research into the implications of this personality type is warranted.

## Introduction

An individual's personality is reflected in their thoughts, emotions, and behavior, which, in turn, influence the health of the person [1]. In recent years, Type D personality has been linked to a wide range of adverse health outcomes [2]–[4]. People with Type D personality tend to have negative emotions towards themselves and others, known as *negative affectivity* (NA). Furthermore, these people are generally afraid of being criticized and rejected by others, so they tend to experience difficulty expressing themselves appropriately in social situations. This results in *social inhibition* (SI) [2], [5]. Several studies of heart disease patients have found that people with a Type D personality have higher cardiac morbidity and higher mortality rates compared with patients with other personality types [3], [5]. This concept has been applied not only to patients with specific diseases but also to the general population, suggesting that Type D personality is associated with poor physical health [4], [6], [7]. A previous study reported that Type D personality can change after severe life events like cardiac surgery [8]. However, Type D personality is considered to be a relatively stable, non-psychopathological character trait, distinct from mental illnesses such as depression [9]–[12]. Thus, researchers have investigated the relationship between Type D personality and various psychological problems, including depression, anxiety, and posttraumatic stress disorder [4], [11], [13], [14]. Type D personality is further associated with work-related problems such as an increased rate of sick leave, job stress, and burnout [15], [16].

In terms of health care utilization, reports indicate that patients with Type D personality rarely receive regular health check-ups [17] or treatment [18]. This could be related to the SI component of Type D personality, which may inhibit these people from seeking adequate care. Then, they tend to be a vulnerable social group. In Japan and other industrialized countries, the burgeoning elderly population is a growing social concern, necessitating efficient and effective social and medical support for the elderly. It is likely that studies on the health effects of Type D personality will be useful in planning appropriate delivery of social/medical resources. However, most studies to date have focused on middle-aged individuals (to our knowledge, the highest average age of the participants in previous studies was 54.2 years) [4], [19], which does not address the need to better understand the elderly population. Further, studies of Type D personality tend to focus on specific at-risk sub-populations, and not the general population. Finally, it appears that most of these studies have been conducted in Western countries [4]. Since the relevant psychosocial concepts are culturally contingent, it is helpful to confirm the health effects in another context. For instance, in Western studies, individuals with Type-A characteristics have been found to be prone to myocardial infarction [20], [21], while Japanese studies have not demonstrated an increased risk of coronary heart disease in this personality type [22], [23].

This study seeks to evaluate the health effects of Type D personality among a general population of elderly people in Japan, using the construct *psychological distress* to represent mental health, and *self-rated health* (SRH) to represent physical health.

## Methods

### Participants

Data were obtained from the Okayama Mental Health Survey of Elderly People, a cross-sectional complete community survey conducted in the Okayama Prefecture, located in the western part of Japan. In August 2010, the Prefectural Government conducted a postal survey of all residents aged 65 and over (*n = *21232) in the three municipalities. Participants were not given any monetary compensation for their involvement, and privacy was ensured by using an anonymous survey (we printed personal identifiers on each questionnaire and used personal data solely to issue a reminder to non-respondents). We received 13929 responses, representing a response rate of 65.6%. We excluded respondents with missing values on the measures related to Type D personality, psychological distress, perceived general health, sex, or age, and 9759 participants were included in the analysis.

A thorough explanation of the aim of the survey was given on the cover of the questionnaire. If residents did not agree to participate in this survey, they could freely choose not to respond without any consequences. Therefore, we considered the receipt of a completed questionnaire to indicate informed consent. The investigators obtained the data from the Okayama Prefectural Government after the removal of personal identifiers. This epidemiological study was reviewed and approved by the Ethics Committee of the Okayama University Graduate School of Medicine, Dentistry and Pharmaceutical Sciences.

### Measures

Type D personality was assessed using the 14-item Type D Personality Scale (DS14) [5]. Participants were asked to rate their responses on a 5-point Likert-type scale (from 0  =  *false* to 4 = *true*). DS14 contains two subscales: negative affectivity (7 items; range 0–28) and social inhibition (7 items; range 0–28). We defined participants with Type D personality as those with scores of greater than 10 on both subscales (i.e., NA & SI) [2], [24].

Psychological distress was evaluated using the Kessler Psychological Distress scale (K6). This instrument has 6 items, and responses are given on a 5-point Likert-type scale (from 0 = *none of the time* to 4 = *all of the time*, total score ranges from 0–24) [25]. The K6 was used to screen participants for mood and anxiety disorders according to the criteria of the Diagnostic and Statistical Manual of Mental Disorders, fourth edition (DSM-IV) [26]. A previous study from Japan [27] used a score of more than 5 on the K6 to indicate psychological distress (sensitivity 100%, specificity 68.7%). In addition to this, we used a cut-off point of >13 (sensitivity 64.7%, specificity 97.3%) [27] to assess severe psychological distress [28]. In previous studies [26], [27], the K6 has been found to be an effective screening method for psychological distress, with results that are as reliable as those of other assessments such as the K10, the Depression and Suicide Screen (DSS), the Center for Epidemiologic Studies Depression Scale (CES-D), and the General Health Questionnaire-12 (GHQ-12). The K6 has also been used to predict suicidal behavior during the past year [29].

The perceived general health of participants was evaluated via one questionnaire item, as follows: “Would you say that in general your health is excellent, very good, good, fair, or poor?” From this item, we created a dichotomous physical health outcome measure (we equated a response of ‘fair’ or ‘poor’ with poor health). Previous studies have found that a poor SRH is a strong predictor of mortality [30], [31].

We identified the following covariates which could act as confounding factors: sex, age (continuous), smoking status (never/former vs. current), frequency of alcohol consumption, overweight status, educational attainment, socioeconomic status, and number of cohabiters. Overweight status was based on body mass index, which was calculated from the height and weight data provided in the questionnaire. An overweight participant was defined as someone with 25 or more kg/m^2^, according to the guidelines of the Japan Society for the Study of Obesity. Frequency of alcohol consumption was divided into four categories: never, 1–3 times/month, 1–6 times/week, and every day. Educational attainment was divided into three categories: junior high school, high school, and college or higher (these divisions took into account historical differences in access to higher education). Socioeconomic status was assessed subjectively by a visual analogue scale (1 = affluent, 9 = disadvantaged), and answers were ranked as *high* (1–4), *middle* (5), *lower middle* (6–8), and *low* (9) according to the distributions. Number of cohabiters was divided into four categories: 1 person (alone), 2, 3, and 4 persons or more.

### Statistical analyses

We first tested for linear trends indicating associations between levels of the Type D subscales (NA & SI) and each health outcome. We then used a logistic regression analysis to further examine associations between Type D personality, psychological distress, and poor SRH, with participants stratified by sex and age group (65–74 y/>75 y). A crude analysis was carried out (Crude Model), and we calculated odds ratios (ORs) and 95% confidence intervals (CIs) for each health outcome. We then adjusted our analysis for the following covariates: sex (only for the age-stratified analysis), age, smoking status, frequency of alcohol consumption, overweight status, educational attainment, socioeconomic status, and number of cohabiters (Adjusted Model). Finally, we imputed data that was missing from the DS14 using the multiple imputation method (Multiple Imputation by Chained Equations: MICE), created five complete datasets, analyzed each dataset, and pooled the results (Imputation). In MICE, all of the covariates were used as independent variables, and each of the DS14 items as an ordinary dependent variable was filled up.

In a sensitivity analysis, we changed the cut-off for K6 scores to 13 to evaluate severe psychological distress. To determine the independent effects of NA and SI, ORs for each health outcome were calculated according to the following groups: NA<10, SI ≥ 10 (i.e., SI+); NA ≥ 10, SI<10 (i.e., NA+); and NA≥10, SI≥10 (i.e., Type D), with a reference of NA<10 and SI<10 (i.e., NA-SI-).

All statistical analyses were carried out using STATA/SE 11.1 (StataCorp, College Station, TX, USA). The level of significance was set at *p*<.05 (two-sided).

## Results

Demographic characteristics and the frequency of Type D personality are shown in Table 1. We found 4508 participants with Type D personality (46.2%), with no substantial difference in prevalence between sexes. In both sexes, we observed a significant dose-response relationship between Type D personality traits and health outcome, with higher levels of NA and SI corresponding to a higher proportion of psychological distress and poor SRH (Table 2).

**Table 1:** Demographic characteristics of participants, Japan, 2010.

	Men (*n* = 4000)	Women (*n* = 5759)
Characteristics	Number (%)	Number (%)
Age: mean [SD]	75.9 [6.9]	76.8 [7.5]
Smoking status		
Never/Former	3147 (78.7)	5240 (91.0)
Current	716 (17.9)	99 (1.7)
Information missing	137 (3.4)	420 (7.3)
Frequency of alcohol consumption		
Never	1307 (32.7)	4138 (71.9)
1–3 times/month	445 (11.1)	675 (11.7)
1–6 times/week	880 (22.0)	528 (9.2)
Every day	1344 (33.6)	177 (3.1)
Information missing	24 (0.6)	241 (4.2)
Body mass index (kg/m^2^)		
Normal (<25)	3203 (80.1)	4529 (78.6)
Overweight (≥25)	686 (17.2)	965 (16.8)
Information missing	111 (2.8)	265 (4.6)
Educational attainment		
Junior high school	1811 (45.3)	2416 (42.0)
High school	1619 (40.5)	2541 (44.1)
College or more	440 (11.0)	492 (8.5)
Information missing	130 (3.3)	310 (5.4)
Socioeconomic status		
High	468 (11.7)	528 (9.2)
Middle	1849 (46.2)	2755 (47.8)
Lower middle	1139 (28.5)	1569 (27.2)
Low	339 (8.5)	549 (9.5)
Information missing	205 (5.1)	358 (6.2)
Number of cohabiters		
1 person (alone)	450 (11.3)	1232 (21.4)
2 persons	1717 (42.9)	1882 (32.7)
3 persons	625 (15.6)	933 (16.2)
4 persons or more	1079 (27.0)	1462 (25.4)
Information missing	129 (3.2)	250 (4.3)
Non-Type D personality	2159 (54.0)	3092 (53.7)
Type D personality	1841 (46.0)	2667 (46.3)

SD: standard deviation.

**Table 2:** Distribution of psychological distress^a^ and poor self-rated health^b^ by levels of Type D subscales, Japan, 2010.

	Men			Women		
	Total	Psychological distress (%)	Poor self-rated health (%)	Total	Psychological distress (%)	Poor self-rated health (%)
	4000	1463 (36.6)	1133 (28.3)	5759	2485 (43.2)	1614 (28.0)
Negative affectivity c						
0/0	341	8 (2.4)	44 (12.9)	378	20 (5.3)	43 (11.4)
1/1–2	105	8 (7.6)	12 (11.4)	388	45 (11.6)	57 (14.7)
2–3/3–5	321	23 (7.2)	54 (16.8)	719	131 (18.2)	121 (16.8)
4–6/6–8	544	94 (17.3)	114 (21.0)	827	250 (30.2)	163 (19.7)
7–10/9–12	773	226 (29.2)	187 (24.2)	1226	557 (45.4)	328 (26.8)
11–16/13–18	1453	728 (50.1)	473 (32.6)	1794	1118 (62.3)	659 (36.7)
17–28/19–28	463	376 (81.2)	249 (53.8)	427	364 (85.3)	243 (56.9)
P for trend		<.001	<.001		<.001	<.001
Social inhibition						
0	118	11 (9.3)	12 (10.2)	157	19 (12.1)	20 (12.7)
1–3	340	35 (10.3)	51 (15.0)	458	82 (17.9)	7.3 (15.9)
4–7	599	105 (17.5)	94 (15.7)	915	241 (26.3)	152 (16.6)
8–11	870	264 (30.3)	214 (24.6)	1302	483 (37.1)	308 (23.7)
12–15	1163	501 (43.1)	342 (29.4)	1727	875 (50.7)	521 (30.2)
16–21	755	427 (56.6)	335 (44.4)	993	628 (63.2)	428 (43.1)
22–28	155	120 (77.4)	85 (54.8)	207	157 (75.9)	112 (54.1)
P for trend		<.001	<.001		<.001	<.001

a Psychological distress denotes K6 score of 5 or higher.

b Poor self-rated health denotes that participant answered either "Fair" or "Poor."

c Cut-off for categories are different between men and women.

In terms of the associations between Type D personality and each health outcome, ORs and 95% CIs are shown in Table 3. Regardless of sex or age stratification, Type D personality was consistently and significantly associated with a higher risk of psychological distress and poor SRH, compared with subjects without Type D. While these associations were of a similar magnitude in both sexes, we found younger participants (65–74 y) to have higher ORs (Adjusted Model, psychological distress; OR: 5.80, 95% CI: 4.96–6.78, poor SRH; OR: 2.84, 95% CI: 2.38–3.38) than participants over 75 (Adjusted Model, psychological distress; OR: 4.54, 95% CI: 3.96–5.19, poor SRH; OR: 2.05, 95% CI: 1.79–2.34). These results were unchanged even when using MICE, suggesting that the ORs significantly increased in all stratified groups (Imputation). (See Table S1 for the demographic characteristics for the 960 participants whose missing data was imputed).

**Table 3:** Odds ratios for psychological distress and poor self-rated health associated with Type D personality, Japan, 2010.

	Psychological distress a			Poor self-rated health b		
	Crude Model	Adjusted Model c	Imputation c	Crude Model	Adjusted Model c	Imputation c
	OR (95% CI)	OR (95% CI)	OR (95% CI)	OR (95% CI)	OR (95% CI)	OR (95% CI)
*Men*						
Non-type D	reference	reference	reference	reference	reference	reference
Type D	5.58 (4.85–6.43)	5.55 (4.74–6.50)	5.43 (4.66–6.34)	2.55 (2.21–2.94)	2.25 (1.91–2.64)	2.26 (1.94–2.64)
*Women*						
Non-type D	reference	reference	reference	reference	reference	reference
Type D	4.93 (4.40–5.52)	4.71 (4.12–5.38)	4.54 (4.00–5.16)	2.68 (2.38–3.02)	2.36 (2.05–2.72)	2.32 (2.03–2.67)
*65*–*74y*						
Non-type D	reference	reference	reference	reference	reference	reference
Type D	6.14 (5.34–7.07)	5.80 (4.96–6.78)	5.73 (4.93–6.68)	3.07 (2.62–3.59)	2.84 (2.38–3.38)	2.83 (2.39–3.36)
*75y+*						
Non-type D	reference	reference	reference	reference	reference	reference
Type D	4.56 (4.08–5.12)	4.54 (3.96–5.19)	4.34 (3.82–4.94)	2.40 (2.14–2.69)	2.05 (1.79–2.34)	2.04 (1.79–2.32)

CI: confidence interval, OR: odds ratio.

a Psychological distress denotes K6 score of 5 or higher.

b Poor self-rated health denotes that participant answered either "Fair" or "Poor."

c Adjusted for age, sex, smoking status, frequency of alcohol consumption, overweight status, educational attainment, socioeconomic status, and number of cohabiters.

The K6 cut-off value of 13 or more was used to assess severe psychological distress. In all stratification groups (with one exception in Adjusted Model among men), ORs were higher than the results for psychological distress (cut-off value of 5 or more) (Table 4). The magnitudes for psychological distress were relatively uniform across sex and age groups (i.e., 4–5 times higher risks). In contrast, for severe psychological distress younger elderly showed strong relationships (OR: 9.92, 95% CI: 5.74–17.12) compared with that of older elderly (OR: 4.62, 95% CI: 3.45–6.17) in Adjusted Model. Further, when we separately analyzed NA and SI (Table 5), we found that NA had a stronger effect on health outcomes than SI. This pattern was clearer for psychological distress than for poor SRH. Notably, even among the non-Type D participants (based on conventional classification [i.e., SI+ or NA+]), all of ORs were significantly high for both psychological distress and poor SRH compared with NA-SI- group.

**Table 4:** Odds ratios for severe psychological distress^a^ associated with Type D personality, Japan, 2010.

	Crude Model	Adjusted Model b	Imputation b
	OR (95% CI)	OR (95% CI)	OR (95% CI)
*Men*			
Non-type D	reference	reference	reference
Type D	6.51 (4.46–9.51)	5.16 (3.41–7.81)	5.68 (3.80–8.51)
*Women*			
Non-type D	reference	reference	reference
Type D	6.18 (4.75–8.02)	6.09 (4.42–8.40)	5.77 (4.26–7.82)
*65*–*74y*			
Non-type D	reference	reference	reference
Type D	9.64 (6.14–15.13)	9.92 (5.74–17.12)	10.39 (6.05–17.83)
*75y+*			
Non-type D	reference	reference	reference
Type D	5.26 (4.11–6.73)	4.62 (3.45–6.17)	4.62 (3.51–6.08)

CI: confidence interval, OR: odds ratio

a Severe psychological distress denotes a K6 score of 13 or higher.

b Adjusted for age, sex, smoking status, frequency of alcohol consumption, overweight status, educational attainment, socioeconomic status, and number of cohabiters.

**Table 5:** Odds ratios for psychological distress and poor self-rated health associated with each component of Type D personality, Japan, 2010.

	Psychological distress a			Poor self-rated health b		
	Crude Model	Adjusted Model c	Imputation c	Crude Model	Adjusted Model c	Imputation c
	OR (95% CI)	OR (95% CI)	OR (95% CI)	OR (95% CI)	OR (95% CI)	OR (95% CI)
NA-,SI-	reference	reference	reference	reference	reference	reference
SI+	2.05 (1.76–2.39)	2.02 (1.69–2.40)	1.96 (1.66–2.32)	1.78 (1.52–2.08)	1.71 (1.43–2.04)	1.66 (1.40–1.97)
NA+	5.49 (4.62–6.53)	5.90 (4.84–7.20)	5.86 (4.84–7.08)	2.16 (1.80–2.61)	2.18 (1.76–2.70)	2.08 (1.69–2.55)
Type D	9.22 (8.17–10.41)	9.21 (8.00–10.60)	8.92 (7.79–10.22)	3.67 (3.25–4.14)	3.21 (2.79–3.70)	3.14 (2.75–3.60)

NA: negative affectivity, SI: social inhibition, CI: confidence interval, OR: odds ratio

a Psychological distress denotes a K6 score of 5 or higher.

b Poor self-rated health denotes that participant answered either "Fair" or "Poor."

c Adjusted for age, sex, smoking status, frequency of alcohol consumption, overweight status, educational attainment, socioeconomic status, and number of cohabiters.

## Discussion

To our knowledge, the present study was based on the largest sample size among any previous studies on Type D personality. Furthermore, this is the first study about Type D personality in Japanese elderly population. Our findings suggest that Type D personality is associated with an adverse health status among elderly Japanese people, both in terms of mental and physical outcomes. After adjusting for covariates, we found that individuals with Type D personality were at 4–5 times the risk of psychological distress and twice the risk of poor SRH. A stratified analysis by age showed that younger elderly participants (65–74 years) were more strongly affected by Type D personality traits than older elderly participants (>75 years). Multiple imputations did not change the results substantially.

Various studies have explored the association between Type D personality and mental illness [4], [14], [32], and to our knowledge, all of these studies reported adverse associations between Type D personality and mental health status, with ORs ranging from 2.6 to 8.6. Our findings, stratified by sex and age, were comparable to these previous studies (adjusted ORs ranging from 4.5 to 5.8). Various instruments have been developed to evaluate psychological distress and symptoms of depression, such as the CES-D, the WHO Composite International Diagnostic Interview (CIDI), the Perceived Stress Scale (PSS), the Patient Health Questionnaire (PHQ), and the K10. This study differed from previous studies in that it used K6 scoring. However, the K6 is highly comparable to both the K10 and the CES-D for assessing mood and anxiety disorders [27], so this difference is unlikely to produce any difficulties in comparing findings between studies. Although the SRH is one of the most widely used health status assessments globally [33]–[35], we know of no previous studies that investigated the association between Type D personality and the SRH. In the present study, individuals with Type D personality showed significantly higher ORs of poor SRH than individuals with non-Type D, suggesting that Type D personality has a negative influence on physical health status, regardless of the methodology (i.e., subjective exposure and outcome).

In this study, participants aged 65–74 years demonstrated consistently higher ORs for psychological distress and poor SRH compared with participants who were over 75 years of age. The most striking finding was the extremely high OR score for severe psychological distress in individuals with Type D personality who were between 65 and 74. This may be due to the influence of various psychosocial changes that accompany the early stages of aging (approximately age 65): decline in physiological function, the death of friends and peers, retirement and loss of professional identity, the independence of one's children, and the loss of previous social roles. Individuals above 75 years of age may have had more time to acquire and familiarize themselves with coping mechanisms for dealing with these psychosocial changes, resulting in a lower OR. Nevertheless, most of the resources are usually designated to deliver much more for older elderly people rather than younger people in many developed countries including Japan. Although further study is necessary, our findings may provide a new perspective about how to efficiently distribute public services.

In previous European studies [4], [7], [12], [36], the percentage of the general population with Type D personality was between 13% and 38.5% (Mean age range: 10.3–54.2). In contrast, the prevalence of Type D personality in the present study was 46.3%, which is much higher than in previous studies. Indeed, this difference may be mainly due to differences in age groups. Furthermore, differences related to race and cultural background is likely to have an influence. For example, Japanese respondents tend to under-report positively phrased items (they are reverse-coded on our one-dimensional scale) compared with Europeans and Americans, causing a bias towards higher scores in Japanese samples [37]. It is possible that a similar tendency affected the responses to the DS14 questionnaire used in the present study. Notably, in the previous studies from Korea and China [38], [39], the proportions of Type D personality among healthy controls were 31.2% and 31.9%, respectively, which are comparable to the European studies. In the Korean study by Lin at al. [38], however, some SI items in the original version were unfamiliar to Koreans, therefore two original items were replaced with other items. A larger and prospective future study may be necessary to show that DS14 is applicable to the Japanese setting with good validity and reliability.

Recent studies might have a possibility of misunderstanding as to the structure of Type D personality, with the categories of Type D and non-Type D made based on selective criteria [40]. Furthermore, several consecutive studies have reported null findings regarding the association of Type D personality with mortality [41]–[43] and other health outcome [44]. We considered these findings when planning the current study, and thus chose to separate the components of NA and SI in our analysis. As a result, NA has a relatively stronger effect on health than SI, particularly in terms of psychological distress. Our findings are in agreement with the classification of the basic characteristics of personality, namely the five-factor model [45]–[47], which shows strong correlations between neuroticism and types of psychological distress, such as depression. In addition, we need to pay attention that there might be considerable risks even in a non-Type D categories according to conventional classifications. It may also suggest that the separate evaluation of NA and SI could reveal additional risks among vulnerable groups.

Our study has several limitations. First, there is a possibility of common-method bias. Although previous studies have repeatedly shown associations between Type D personality and depression or psychological distress [4], [11], [13], [14], the influence of similar items, including those addressed in the DS14 and the K6 questionnaire has to be discussed. We can evaluate this influence in a partial way because, while the NA subscale shared some with items with the K6, there is no overlap in SI subscales. Furthermore, the SRH does not share items with the DS14. Hence, the consistency of our findings across exposures and outcomes would have some validity. Although some elements of Type D personality and depression do overlap, previous studies using factor analysis have found that the Type D personality scale and measures of depressive symptoms are different and distinct [9], [32]. A second limitation of our study is related to the assessment of mental health, and we should be aware of the possibility that participants with mental illness/cognitive deterioration did not complete the questionnaire accurately. Although information about the depression/cognitive function of our participants was unavailable, future studies are warranted by assessing comorbidity, focusing on depression and dementia, rather than psychological distress. Third, because of the cross-sectional design of our study, we cannot rule out the possibility of reverse causation. Thus, careful interpretation is necessary. The DS14 evaluates personality based on questions that measure stable long-term characteristics. The K6, on the other hand, specifies a concrete time period (the previous 30 days) and the SRH asks the present status of general health. Thus, the temporal relationship between exposure (DS14) and self-reported mental/physical outcomes was determined. A fourth limitation is selection bias, whereby participants with Type D personality and poor health outcomes may have opted not to participate in the study. This could lead to an underestimation of the present findings.

In conclusion, the present study shows that Japanese elderly people with Type D personality have an enhanced risk of psychological distress as well as poor SRH. The effect of personality on health is likely to be culturally contingent, and this is the first study to examine the health effects of Type D personality in a Japanese elderly population. In addition, this is the first study to demonstrate the validity of previous findings for this specific group. As developed countries face an increasingly elderly population, and consequently, an increasing need for various types of healthcare, the present findings may aid the development of efficient social services. To this end, an enhanced understanding of connections between the mental and physical health of the elderly is essential.

## Supporting Information

Table S1 **Demographic characteristics for the 960 participants whose missing data was imputed, Japan, 2010.**
(PDF)
