# Supplementary material for: Type D Personality Is Associated with Psychological Distress and Poor Self-Rated Health among the Elderly: A Population-Based Study in Japan
Source: PLoS One. 2013 Oct 17;8(10):e77918. doi: 10.1371/journal.pone.0077918 (PMC3798570; doi:10.1371/journal.pone.0077918)
Supplement: Table S1 — Demographic characteristics for the 960 participants whose missing data was imputed, Japan, 2010. (PDF) [file pone.0077918.s001.pdf]

Table S1. Demographic characteristics for the 960 participants whose missing data was imputed, Japan, 2010

| Characteristics | Men (*n* = 385) Number (%) | Women (*n* = 575) Number (%) |
|---|---|---|
| Age: mean [SD] | 76.6 [6.9] | 78.2 [7.1] |
| Smoking status | | |
| Never/Former | 319 (82.9) | 571 (99.3) |
| Current | 66 (17.1) | 4 (0.7) |
| Frequency of alcohol consumption | | |
| Never | 135 (35.1) | 460 (80.0) |
| 1–3/month | 50 (13.0) | 63 (11.0) |
| 1–6/week | 70 (18.2) | 30 (5.2) |
| Every day | 130 (33.8) | 22 (3.8) |
| Body mass index ($kg/m^2$) | | |
| Normal (<25) | 312 (81.0) | 478 (83.1) |
| Overweight (≥25) | 73 (19.0) | 97 (16.9) |
| Educational attainment | | |
| Junior high school | 226 (58.7) | 285 (49.6) |
| High school | 129 (33.5) | 254 (44.2) |
| College or more | 30 (7.8) | 36(6.3) |
| Socioeconomic status | | |
| High | 31 (8.1) | 48 (8.4) |
| Middle | 204 (53.0) | 290 (50.4) |
| Lower middle | 114 (29.6) | 177 (30.8) |
| Low | 36 (9.4) | 60 (10.4) |
| Number of cohabiters | | |
| 1 person (alone) | 46 (12.0) | 140 (24.4) |
| 2 persons | 166 (43.1) | 184 (32.0) |
| 3 persons | 62 (16.1) | 92 (16.0) |
| 4 persons or more | 111 (28.8) | 159 (27.7) |

SD: standard deviation
